# Neuroprotection: Rescue from Neuronal Death in the Brain 2.0

**DOI:** 10.3390/ijms24065273

**Published:** 2023-03-09

**Authors:** Bae Hwan Lee

**Affiliations:** 1 Department of Physiology, Yonsei University College of Medicine, Seoul 03722, Republic of Korea; bhlee@yuhs.ac; 2 Department of Medical Science, Brain Korea 21 Project, Yonsei University College of Medicine, Seoul 03722, Republic of Korea

The brain is vulnerable to endogenous or exogenous injuries [1]. Neuronal death from brain injuries may deteriorate neurological functions, and they often result in neurodegenerative diseases. Therefore, protection against neuronal death in the brain is very important for preserving normal functions.

Following the previous Special Issue, this issue aimed to identify the mechanisms of neuroprotection and ascertain therapeutic strategies for functional recovery from neurological deficits. In this Special Issue, ten papers were published, including six original articles and four review papers related to neural injury and neuroprotection.

Traumatic brain injury (TBI) mostly results from insults or accidents and induces immediate damage to the brain. Vascular endothelial growth factor (VEGF) is known to play a crucial role in TBI. Genovese et al. [2] reported that VEGF expression was increased following motor deficits induced by TBI. Bevacizumab, a VEGF inhibitor, improved motor deficits and also decreased the VEGF levels, brain edema, and neuroinflammation. The integrity of the blood–brain barrier was preserved by bevacizumab. The results suggest that bevacizumab has an neuroprotective effect on TBI.

An oxidative injury may induce neurodegenerative diseases [3]. A redox imbalance between prooxidant and antioxidant agents induces the formation of free radicals such as reactive oxygen species (ROS). In a review paper, Lee et al. [4] delineated the crosstalk between neuron and glial cells to maintain antioxidant defense mechanisms. In particular, they showed that an oxidative injury induces morphological and molecular changes in glial cells and regulates neuronal activities under oxidative stress.

Hemoglobin (Hb) is an oxygen transport protein in red blood cells and a tetramer made of four globin monomers, usually two Hb alpha subunits (Hb-α) and two Hb beta subunits (HB-β). Each monomeric chain has a heme moiety for O_2_ transport. It has been shown that Hb-α is also expressed in neurons. Lu et al. [5] examined the regulation of Hb-α and found that neuronal Hb-α in the hippocampus and cortex decreased with age in mice and lentivirus CRISPR interference-based Hb-α knockdown (Hb-α CRISPRi KD) in the hippocampus worsened hypoxia and increased the levels of hypoxia-inducible factor-1α and pro-apoptotic factors. These results imply that neuronal Hb-α plays a crucial role in the regulation of oxygenation and neuroprotection in the brain.

Nimodipine is a lipophilic L-type calcium channel blocker and a potent cerebral vasodilator. Hohmann et al. [6] investigated the neuroprotective effect of nimodipine on excitotoxic injuries in organotypic hippocampal slice cultures (OHSCs) and found that nimodipine reduced the impact of the injury in OHSCs when they were co-applied with NMDA, indicating the neuroprotective effect on excitotoxic injuries in the hippocampus.

Neuronal apoptosis induced by hydrogen peroxide (H_2_O_2_) has been shown to be critical to the development of Alzheimer’s disease (AD) and other neurodegenerative diseases. One of the causes for apoptosis is oxidative stress. Apolipoprotein E (ApoE) has three major isoforms, ApoE2, ApoE3, and ApoE4. Gao et al. [7] studied the neuroprotective effects of ApoE isomers on H_2_O_2_-induced apoptosis using human iPSC-derived neurons and reported that ApoE2 and ApoE3 demonstrated protection against apoptosis through the signaling pathway, activating Akt and inhibiting FoxO3a/Bim, but ApoE4 did not. These findings suggest that ApoE may play isoform-specific roles in neurodegenerative diseases related to ROS-induced apoptosis.

Parkinson’s disease (PD) is one of the most well-known neurodegenerative disease. In relation to PD, Behl et al. [8,9,10] published three review papers. Firstly, Behl et al. [8] delineated the role of peroxisome proliferator-activated receptors (PPARs), ligand-directed transcription factors, in PD. Secondly, Behl et al. [9] described the metabolism of redox-active metals such as iron (Fe) and copper (Cu) in the central nervous system (CNS) and their role and disrupted balance in PD. Thirdly, Behl et al. [10] reviewed the neuroprotective role of numerous neuropeptides such as substance P, ghrelin, neuropeptide Y, neurotensin, pituitary adenylate cyclase-activating polypeptide (PACAP), nesfatin-1, and somatostatin in PD. In an original article, Haque et al. [11] investigated the role of the proton-activated G protein-coupled receptor 4 (GPR4) in a 1-methyl-4-phenyl-1,2,3,6-tetrahydropyridine (MPTP)-induced mouse model of PD and found that a selective GPR4 antagonist reduced dopaminergic cell death, improved motor function, decreased pro-apoptotic marker Bax protein levels, and increased anti-apoptotic marker bcl-2 protein levels in the substantia nigra pars compacta and striatum. These results suggest the neuroprotective effects of GPR4, which is involved in caspase-mediated apoptosis in the substantia nigra pars compacta and striatum.

The visual system of experimental animals has been used to study the degenerative and regenerative capability of CNS neurons. Using an animal model of intraorbital optic nerve transection (IONT), Gallego-Ortega et al. [12] investigated the neuroprotective effects of 7,8-dihydroxiflavone (DHF) and found that IONT induced the functional impairment of the retinal ganglion cells (RGCs), and such a deficit was prevented by DHF in various types of RGCs. These findings imply the neuroprotective role of DHF in the optic nerve.

It has been known that many different types of injury affect brain functions, leading to complex mechanisms of neurological disorders. Obtaining a better understanding of the pathophysiological mechanisms of neuronal death and neuroprotection may contribute to the diagnosis and therapy of deteriorated brain functions, thus preserving normal functions.
